# *PKD1* Mutation Is a Biomarker for Autosomal Dominant Polycystic Kidney Disease

**DOI:** 10.3390/biom13071020

**Published:** 2023-06-21

**Authors:** Tomoki Kimura, Haruna Kawano, Satoru Muto, Nobuhito Muramoto, Toshiaki Takano, Yan Lu, Hidetaka Eguchi, Hiroo Wada, Yasushi Okazaki, Hisamitsu Ide, Shigeo Horie

**Affiliations:** 1 Department of Urology, Juntendo University Graduate School of Medicine, Tokyo 113-8431, Japan; 2 Department of Advanced Informatics for Genetic Diseases, Juntendo University Graduate School of Medicine, Tokyo 113-8431, Japan; 3 Department of Urology, Juntendo University Nerima Hospital, Tokyo 177-8521, Japan; 4 Human Disease Models, Institute of Laboratory Animals, Tokyo Women’s Medical University, Tokyo 162-8666, Japan; 5 Diagnostics and Therapeutics of Intractable Diseases, Intractable Disease Research Center, Juntendo University Graduate School of Medicine, Tokyo 113-8431, Japan; 6 Department of Public Health, Juntendo University Graduate School of Medicine, Tokyo 113-8431, Japan; 7 Department of Digital Therapeutics, Juntendo University Graduate School of Medicine, Tokyo 113-8431, Japan

**Keywords:** ADPKD, analysis of germline mutations, biomarkers, *PKD1* mutation, predicting renal prognosis

## Abstract

Background: Autosomal dominant polycystic kidney disease (ADPKD) occurs in 1 in 500–4000 people worldwide. Genetic mutation is a biomarker for predicting renal dysfunction in patients with ADPKD. In this study, we performed a genetic analysis of Japanese patients with ADPKD to investigate the prognostic utility of genetic mutations in predicting renal function outcomes. Methods: Patients clinically diagnosed with ADPKD underwent a panel genetic test for germline mutations in *PKD1* and *PKD2*. This study was conducted with the approval of the Ethics Committee of Juntendo University (no. 2019107). Results: Of 436 patients, 366 (83.9%) had genetic mutations. Notably, patients with *PKD1* mutation had a significantly decreased ΔeGFR/year compared to patients with *PKD2* mutation, indicating a progression of renal dysfunction (−3.50 vs. −2.04 mL/min/1.73 m^2^/year, *p* = 0.066). Furthermore, *PKD1* truncated mutations had a significantly decreased ΔeGFR/year compared to *PKD1* non-truncated mutations in the population aged over 65 years (−6.56 vs. −2.16 mL/min/1.73 m^2^/year, *p* = 0.049). Multivariate analysis showed that *PKD1* mutation was a more significant risk factor than *PKD2* mutation (odds ratio, 1.81; 95% confidence interval, 1.11–3.16; *p* = 0.020). Conclusions: The analysis of germline mutations can predict renal prognosis in Japanese patients with ADPKD, and *PKD1* mutation is a biomarker of ADPKD.

## 1. Introduction

Polycystic kidney disease is a disorder characterized by the development of numerous bilateral kidney cysts. It is classified into autosomal dominant polycystic kidney disease (ADPKD) and autosomal recessive polycystic kidney disease (ARPKD) according to the type of inheritance [1]. ADPKD is estimated to have an incidence of approximately 1 in 500–4000 people worldwide and occurs in both sexes, with no sex differences [1,2,3,4,5].

With age, numerous cysts develop progressively and enlarge bilaterally in the kidneys, which is accompanied by decreased renal function [3]. Most patients are asymptomatic until the age of 30–40 years, after which renal function gradually declines, and approximately half of them develop end-stage kidney disease (ESKD) by the age of 60–70 years [1,5]. However, phenotypes such as clinical symptoms appear in adulthood. In addition to the autosomal manifestation of inheritance, the second-hit theory is thought to explain why phenotypes differ even in the same family [6].

The two representative mutations in ADPKD are *PKD1* and *PKD2*, which encode polycystin 1 (PC1) and polycystin 2 (PC2), respectively [7,8,9]. Approximately 85% of patients with ADPKD have a *PKD1* mutation, whereas the remaining 15% have a *PKD2* mutation [10]. The significance of studying the genetic background of patients with ADPKD includes not only the diagnostic aspect, but also the predictive aspect of renal prognosis. Patients with *PKD1* mutations have been shown to have a poorer renal prognosis than those with *PKD2* mutations; in addition, patients below 55–58 years of age with a family history of ESKD are more likely to have *PKD1* mutations, and those above 68–70 years of age with a family history of ESKD are more likely to have *PKD2* mutations [11]. Furthermore, the truncated *PKD1* mutation that results in a major change in protein structure has been reported to be associated with a worse renal prognosis, with a median age of 55.6 years, while that for the non-truncated mutation is 67.9 years [12]. As mentioned above, *PKD1* truncated mutations are known to have a faster rate of renal function decline and worse renal prognosis than non-truncated mutations.

Currently, tolvaptan, a vasopressin V2 receptor antagonist, is approved and has been shown to be an effective prophylactic treatment for ADPKD with worsening renal outcomes [13,14]. However, due to the side effects and medication management, it is not generally recommended for use in all patients with ADPKD, and the target population remains controversial.

Blood and urine markers have been reported to be useful for predicting worsening renal function in patients with ADPKD, and, in a previous report, neutrophil gelatinase-associated lipocalin, lipocalin-2 (NGAL), macrophage-colony stimulating factor (M-CSF), and monocyte chemoattractant protein-1 (MCP-1) were useful urinary biomarkers [15,16,17,18,19,20,21,22]. The severity classifications of ADPKD include the Mayo classification and the Predicting Renal Outcome in Polycystic Kidney Disease (PROPKD) score [23,24]. The Mayo classification predicts renal prognosis by correlating this with decreased eGFR through stratification by age and HtTKV (classes 1A–E). In contrast, the PROPKD score is based on (1) sex (0 for females, 1 for males), (2) hypertension (0 for none, 1 for all), (3) urologic events (0 for none, 1 for all), and (4) genetic mutations (*PKD2* mutation: 0, *PKD1* non-truncated mutation: 2, *PKD1* truncated mutation: 4). The median age for ESKD onset has been reported to be 49 years for a score of ≥7, 56.9 years for a score of 4–6, and 70.6 years for a score of 0–3, and the higher the score, the worse the prognosis [24].

Although there have been several reports of genetic mutations in Japanese patients with ADPKD, including ours [7,25,26], they have not been sufficiently investigated as biomarkers on a large scale. Therefore, we performed a large-scale genetic analysis of Japanese patients with ADPKD to investigate the prognostic value of genetic variants for predicting renal outcomes.

This study aimed to establish a database of Japanese patients with ADPKD and analyze information on genetic mutations leading to exacerbations. This may assist in the understanding of the pathophysiology of ADPKD and provide appropriate therapeutic interventions for ADPKD patients.

## 2. Materials and Methods

### 2.1. Study Subjects

We included adult patients who were clinically diagnosed with ADPKD according to Ravine’s criteria [27] between November 2018 and March 2023 and who, after receiving a full explanation of their participation in the study, provided free and voluntary written consent with full understanding. Patients were excluded if they were ineligible due to missing data or missed hospital visits. This study was conducted with the approval of the Juntendo University Ethics Committee (no. 2019107). The exclusion criterion was the determination of inappropriateness to participate in this study by the principal investigator.

### 2.2. Research Methods

(1) Sample Collection

We collected 7 mL of blood from the patients, and an additional 7 mL of blood was collected when the blood cells were cultured prior to extraction for total RNA sequence analysis. This was performed only for the purpose of conducting this study, rather than incidentally when performing the tests necessary for the diagnosis and treatment of the subjects’ own diseases. We collected blood samples every 3 months;

(2) Use of Existing Data and Information

We obtained written consent from the patients for the use of existing blood tests, imaging tests, and other data from medical records in this genetic analysis study. To assess renal function and the progression of renal dysfunction, we used estimated glomerular filtration rate (eGFR) and ΔeGFR/year. The eGFR was calculated as follows: eGFR = 194 × serum Cr-1.094 × age-0.287 (×0.739 if female) [28]. Additionally, the ΔeGFR/year was calculated by creating an approximate curve from the eGFR values measured over time. The cutoff value of ΔeGFR/year was 3.61 mL/min/1.73 m^2^/year [29,30]. Furthermore, the total kidney volume (TKV) was assessed and measured using computed tomography or magnetic resonance imaging. A single urologist performed the TKV measurements to avoid different results from different raters. The TKV was estimated using the ellipsoid volume of revolution method as follows: (π/6 × major diameter × [minor diameter]^2^). In the current study, we used the height-adjusted TKV (HtTKV), which has been shown to correlate with renal function without sex differences [31]. We used the Irazabal equation to calculate future eGFR and estimated the age leading to ESKD (future eGFR < 15 mL/min/1.73 m^2^) (Table 1) [23,32].

Future eGFR = *α* + *β* + *γ*(baseline age)

+ *δ*(baseline eGFR) + *θ*

+ *ε*(years from baseline)

+ *λ*(1 if male, 0 otherwise) (years from baseline) + *μ*(current age)(years from baseline)

+ *σ*(years from baseline);

(3) Genes/Gene Groups to be Analyzed and Analysis Methods

- Targeted Resequencing

In this study, we performed a panel genetic test for germline mutations that targets known causative genes of the target disease and the diseases to be differentiated. Target genes included *PKD1*, *PKD2*, *PKHD1*, *TSC1*, *TSC2*, *PRKCSH*, *SEC63*, *LRP5*, *VHL*, *HNF1B*, *MUC1*, *UMOD*, *OFD1*, and *GANAB*.

We designed primers for target genomic regions using the Ion AmpliSeq^TM^ Designer, performed target enrichment to enrich target DNA fragments by multiplex PCR amplification using Ion Chef, and performed library and template preparation following the manufacturer’s instructions.

Sequencing data were obtained by performing the sequence on bench-top next-generation sequencers such as the Ion S5 Plus or Ion PGM systems;

ii. Sanger Sequencing

We performed gene-specific long PCR for the exon1 region of the *PKD1* mutation that could not be covered by targeted resequencing, and direct sequencing was performed using this as a template;

iii. Copy Number Variation Analysis (Multiplex Ligation-Dependent Probe Assay (MLPA) Method)

The MLPA method was used to detect copy number variations in each exon unit of a gene using the SALSA MLPA probe mix and SALSA MLPA EK1 reagent kit (MRC-Holland). Moreover, a 3500 Genetic Analyzer was used for fragment analysis, and the obtained data were analyzed using the MRC-Holland software. The obtained data were analyzed using MRC-Holland’s coffalyser.net software;

iv. Total RNA Sequence Analysis

We performed total RNA sequence analysis to detect fusion genes, intragenic inversions, splicing abnormalities caused by mutations in deep intron regions, transcriptional repression caused by mutations in promoter regions, and promoter switching, which could not be detected by DNA sequencing.

Total RNA was extracted from peripheral blood using the QIAGEN RNeasy Mini Kit or the QIAamp RNA Blood Mini Kit, following the manufacturer’s instructions. Libraries were prepared using Illumina’s TruSeq Stranded mRNA Library Prep Kit, and sequencing data were obtained using HiSeq4000;

v. Whole-Exome Sequencing Analysis

We performed exome capture and library preparation using SureSelect Human All Exome V6 (58 M) (Agilent), and analysis was performed using an Illumina next-generation sequencer;

vi. Bioinformatics Analysis

We performed data quality checks, mapping, assembly, and mutation detection using FASTQ files obtained using existing pipelines. For known pathological mutations, we used databases such as ClinVar, The PKD Mutation Database, Mutation Database Autosomal Recessive Polycystic Kidney Disease (ARPKD/PKHD1), The Human Gene Mutation Database (HGMD), and other databases to determine pathogenicity. Moreover, for mutations not registered in public databases, pathological mutations were classified according to the ACMG guidelines [33].

Variants of unknown significance (VUS) were classified as pathological mutations according to the ACMG guidelines [33] using software programs such as PANTHER, PROVEAN, MAPP, Align-GVGD, PON-P2, and FATHMM. We analyzed candidate splicing mutations using prediction tools such as the Human Splicing Finder and BDGP (Splice Site Prediction by Neural Network);

vii. Statistical Analysis

We performed analyses to investigate the relationship between pathological variants of causative genes such as *PKD1/PKD2* and annual changes in renal function and TKV. Furthermore, we evaluated the following clinicopathological prognostic factors indicated by a previous study as adjustment factors: sex, age, hypertension by 35 years of age, urologic events by 35 years of age (including cyst infection, gross hematuria, and/or flank pain related to cysts), and urinalysis [24]. We also compared the following categories of pathological genetic variants: (a) among the three causative gene groups (*PKD1*, *PKD2*, and others) and (b) between the two groups of *PKD1* genetic mutations (truncated and non-truncated). *PKD1* truncated and non-truncated mutations were divided into two groups based on the World Health Organization definition of the elderly: those aged 65 years or older and others.

For the genetic analysis, the Mann–Whitney U test was used for comparison between two groups in the subgroup analysis, and the Kruskal–Wallis test was used for comparison between three or more groups. Additionally, we used the chi-square and Fisher’s exact tests as analytical methods to compare the ratios between genetic variants and other variables. For risk factors, parameters associated with decreased renal function were selected as explanatory variables, and multivariate analysis using logistic regression was used to examine the significant differences between the groups. To adjust for patient background, we used matched-pair analysis with propensity score matching. We used the Irazabal equation to calculate future eGFR and estimated the age leading to ESKD (future eGFR < 15 mL/min/1.73 m^2^) [23,32]. Kaplan–Meier survival curves were plotted and compared using the log-rank test. All statistical analyses were performed using EZR (Saitama Medical Center, Jichi Medical University, Saitama, Japan) [34], and statistical significance was defined as *p* < 0.05.

## 3. Results

**Figure 1:**
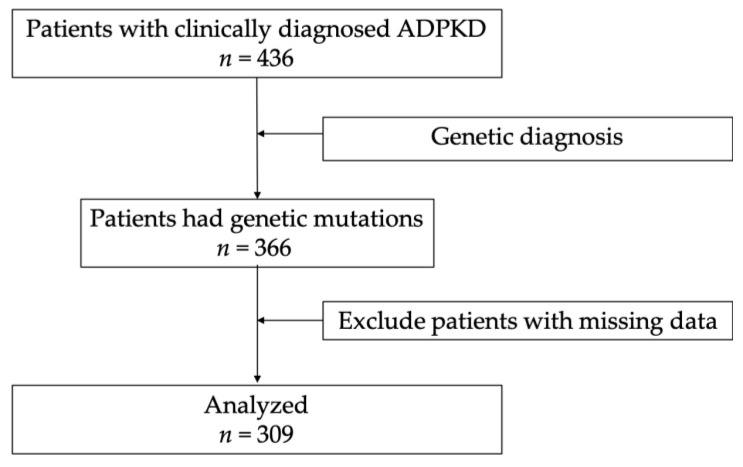
Flow chart of patients with ADPKD. ADPKD: autosomal dominant polycystic kidney disease.

Of the 436 patients clinically diagnosed with ADPKD, 366 (83.9%) had genetic mutations (Figure 1). The genetic mutations identified (*n* = 366) were *PKD1* truncated, *PKD1* non-truncated, *PKD2* truncated, *PKD2* non-truncated, *GANAB* non-truncated, *OFD1* truncated, and *SEC63* non-truncated. Three patients (0.8%) had mutations other than *PKD1* and *PKD2* genetic mutations, as detected by the target gene panel (*GANAB* non-truncated, *OFD1* truncated, and *SEC63* non-truncated, respectively). Within the 363 patients with a genetic mutation of *PKD1* (273 patients, 74.6%) or *PKD2* (90 patients, 24.6%), sixteen patients (4.4%) had CNVs detected by MLPA (Table S1 in Supplement [33,35]).

Table 2 shows that the median age was 48 (41–55) years, the median HtTKV was 748.0 mL (483.3–1002.2 mL), the median ΔeGFR/year was −3.10 mL/min/1.73 m^2^ (−5.69 to −1.0 mL/min/1.73 m^2^), and classes 1A, 1B, 1C, 1D, and 1E of Mayo classification were 19, 103, 121, 54, and 12, respectively.

Furthermore, the number of patients with *PKD1* truncated, *PKD1* non-truncated, *PKD2* truncated, and *PKD2* non-truncated genetic mutations was 139 (45.0%), 86 (27.8%), 68 (22.0%), and 16 patients (5.2%), respectively (Table 2).

A subgroup analysis of ΔeGFR/year was performed on 309 patients with ADPKD who had genetic mutations, after excluding those with missing data (Table 3). The median values for each clinical factor are shown in Table 2. These values were compared between the groups.

**Figure 2:**
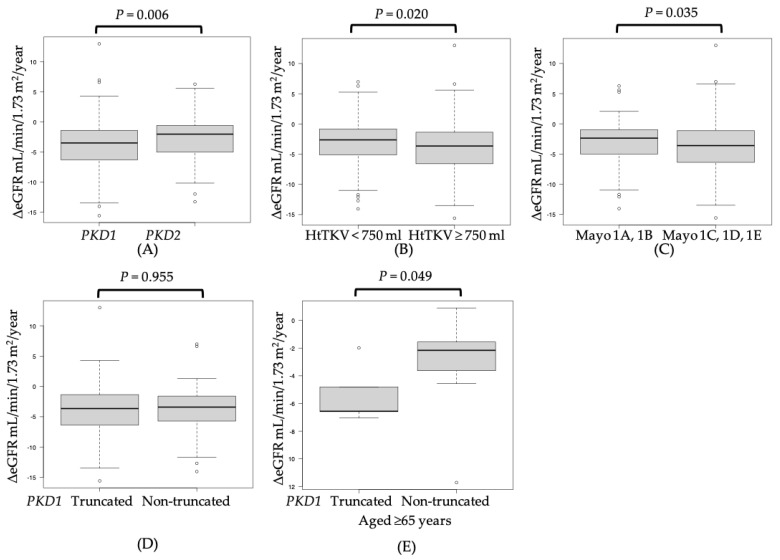
Mann−Whitney analysis of the clinically important factors related to the rate of change in ΔeGFR performed in the subgroup analysis and comparison of ΔeGFR between patients with PKD1 and PKD2 (**A**), HtTKV (**B**), Mayo 1A and 1B and 1C, 1D, and 1E (**C**), PKD1 truncated or non-truncated mutations (**D**), and *PKD1* truncated or non-truncated mutations in the population aged ≥65 years (**E**). *HtTKV*: height-adjusted total kidney volume (mL/m), eGFR: estimated glomerular filtration rate, ΔeGFR/year: represents the 1-year change in eGFR calculated using the least-squares method based on the change in eGFR values before tolvaptan treatment, aged ≥65 years: population aged ≥65 years.

We performed additional analyses for clinically important factors that were related to the rate of change in ΔeGFR in the subgroup analyses. We found that the group of patients with a *PKD1* mutation had a significantly decreased ΔeGFR/year compared to the group of patients with a *PKD2* mutation, indicating the progression of renal dysfunction (−3.50 vs. −2.04 mL/min/1.73 m^2^/year, *p* = 0.066) (Figure 2A). Moreover, the group with a HtTKV ≥ 750 mL had a significantly decreased ΔeGFR/year compared to the group with a HtTKV < 750 mL (−3.65 vs. −2.64 mL/min/1.73 m^2^/year, *p* = 0.020) (Figure 2B). Regarding the Mayo classification using HtTKV and age, patients in groups 1C, 1D, and 1E had a significantly decreased ΔeGFR/year compared to patients in groups 1A and 1B, indicating progression of renal dysfunction (−2.38 vs. −3.61 mL/min/1.73 m^2^/year, *p* = 0.035) (Figure 2C). However, there was no significant difference in ΔeGFR/year between those with truncated and non-truncated *PKD1* mutations in all age groups (−3.65 vs. −3.41 mL/min/1.73 m^2^/year, *p* = 0.955) (Figure 2D). In contrast, in the population older than 65 years, *PKD1* truncated mutations showed a significantly decreased eGFR/year compared to *PKD1* non-truncated mutations (−6.56 vs. −2.16 mL/min/1.73 m^2^/year, *p* = 0.049) (Figure 2E).

Table 4 shows the percentage change in ΔeGFR/year for the following factors with body mass index (BMI), HtTKV, and Mayo classification that were significantly different in the subgroup analysis: *PKD1* or *PKD2* mutations, truncated or non-truncated *PKD1* mutations in patients aged 65 years and older, and truncated or non-truncated *PKD1* mutations in patients aged 65 years and older. There was a significant difference in the Mayo classification ratio between *PKD1* and *PKD2* mutations (*p* = 0.015), whereas none of the ratios for BMI or HtTKV was significantly different.

In the univariate logistic regression analysis, there were no significant differences in sex, hypertension before 35 years of age, or urologic events before 35 years of age as risk factors when ΔeGFR/year > 3.61 mL/min/1.73 m^2^/year was used as the cutoff value. We also found that *PKD1* mutation was a more significant risk factor than *PKD2* mutation (odds ratio (OR), 1.81; 95% confidence interval (CI), 1.08–3.05; *p* = 0.025), and HtTKV ≥ 750 mL was also a significant risk factor (OR, 1.62; 95% CI, 1.03–2.54; *p* = 0.027). Then, in the multivariate logistic regression analysis, *PKD1* mutation was a more significant risk factor than *PKD2* mutation (OR, 1.87; 95% CI, 1.11–3.16; *p* = 0.020), and HtTKV ≥ 750 mL was also a significant risk factor (OR, 1.67; 95% CI, 1.06–2.63; *p* = 0.029). Furthermore, the data were abstracted using matched-pair analysis with propensity score matching to adjust for the background with age, sex, height, BMI, hypertension before 35 years of age, urologic event before 35 years of age, and U-pro (Table S2). Additionally, in the multivariate logistic regression analysis of this data after adjustment on the propensity score, *PKD1* mutation was a more significant risk factor than *PKD2* mutation (OR, 2.44; 95% CI, 1.23–4.82; *p* = 0.011), and HtTKV ≥ 750 mL was also a significant risk factor (OR, 2.58; 95% CI, 1.30–5.13; *p* = 0.007) (Table 5).

In 315 patients, including six dialysis patients for whom ΔeGFR could not be calculated, we used future eGFR to predict age leading to ESKD (future eGFR < 15 mL/min/1.73 m^2^).

**Figure 3:**
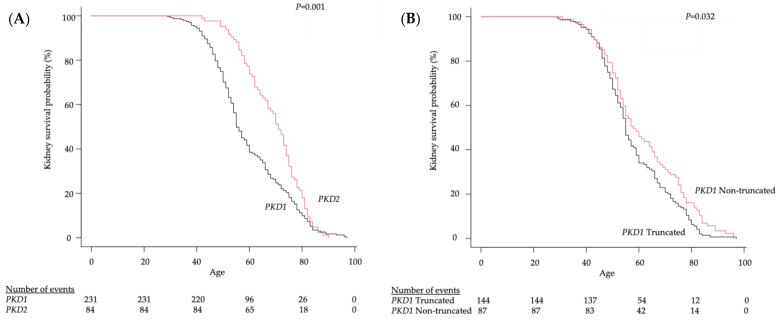
Kaplan–Meier kidney survival plot of the group of patients with a *PKD1* mutation and the group of patients with a *PKD2* mutation (**A**), *PKD1* truncated or non-truncated mutations (**B**).

As shown in Figure 3, the median age at ESKD onset in *PKD1* mutation group was 55 years (95% CI, 54–59 years), and the median age at ESKD onset in the *PKD2* mutation group was 71 years (95% CI, 67–74 years) (*p* = 0.001). Moreover, the median age at ESKD onset in the *PKD1* truncated mutation group was 55 years (95% CI, 54–57 years), and the median age at ESKD onset in the *PKD1* non-truncated mutation group was 58 years (95% CI, 54–65 years) (*p* = 0.032).

In a Kaplan–Meier kidney survival plot, we found that the group of patients with a *PKD1* mutation showed significantly worse kidney survival compared to the group of patients with a *PKD2* mutation, and those with *PKD1* truncated mutations showed significantly worse kidney survival compared to those with *PKD1* non-truncated mutations.

## 4. Discussion

To our knowledge, the present study identifying risk factors for renal function decline in Japanese patients with ADPKD is the largest single-center prospective study in Japan with the largest number of patients. We showed that patients with *PKD1* mutations and increased HtTKV with *PKD1* truncated mutations are expected to have a more rapid progression of renal dysfunction with age than those with non-truncated mutations.

In the present study, of the 436 patients clinically diagnosed with ADPKD, 366 (83.9%) had genetic mutations (Figure 1). Among patients with genetic mutations, 273 (74.6%) carried a *PKD1* mutation, and 90 (24.6%) carried a *PKD2* mutation. The five prior large cohort studies reported the distribution of *PKD1* and *PKD2* mutations in 202 (USA) [36], 700 (France) [35], 220 (Canada) [37], 643 (Italy) [38], and 1119 (USA) [39] patients. Each of these studies reported high detection rates at 89.1%, 89.9%, 84.5%, 80%, and 92.4%, respectively, which do not differ from that observed in the present study.

In this study, although no significant difference was observed in the overall age group in the rate of change of ΔeGFR (−3.65 vs. −3.41 mL/min/1.73 m^2^/year, *p* = 0.955) between the *PKD1* truncated mutation group and non-truncated mutation group (Figure 2D), a significant difference in the rate of change of ΔeGFR was observed in the population aged 65 years and older (−6.56 vs. −2.16 mL/min/1.73 m^2^/year, *p* = 0.049) between these groups (Figure 2E). The median age at ESKD onset in the *PKD1* mutation group was 55 years (95% CI, 54–59 years), and the median age at ESKD onset in the *PKD2* mutation group was 71 years (95% CI, 67–74 years) (*p* = 0.001) (Figure 3A). Moreover, the median age at ESKD onset in the *PKD1* truncated mutation group was 55 years (95% CI, 54–57 years), and the median age at ESKD onset in the *PKD1* non-truncated mutation group was 58 years (95% CI, 54–65 years) (*p* = 0.032) (Figure 3B). Cornec-Le Gall et al. reported that the median age at ESKD onset was 55.6 years (95% CI, 53.6–57.7 years) in the *PKD1* truncated mutation group and 67.9 years (95% CI, 62.4–73.4 years) in the *PKD1* non-truncated mutation group, showing a difference in the *PKD1* non-truncated mutation group, as compared with that in our study [12].

Regarding renal function in ADPKD, the GFR is normal owing to nephron compensation until renal enlargement is marked by numerous cysts. The GFR begins to decline at an average age of approximately 40 years, and the rate of renal function decline increases as renal reserves are reduced [40]. Therefore, the identification of genetic mutations at a young age can help identify patients at high risk of a faster decline in renal function, leading to earlier treatment interventions.

Tolvaptan, a vasopressin V2 receptor inhibitor used for the treatment of ADPKD, has been shown to inhibit renal volume increase and renal function decline [41]. Moreover, earlier induction is associated with a lower renal prognosis [13,30,41,42]. Additionally, the higher the volume of HtTKV, the faster the rate of renal function decline and the worse the renal prognosis [17,18,40,43,44,45]. In the present study, significant differences were observed in the two groups divided by an HtTKV cutoff value of 750 mL (−3.65 vs. −2.64 mL/min/1.73 m^2^/year, *p* = 0.020) (Figure 2B). A previous study reported that an algorithm using age and eGFR can predict the rapid progression of renal function and identify patients who can be treated with tolvaptan [20]. This suggests that patients with *PKD1* truncated mutations require early and appropriate treatment. However, there have been some reports on tolvaptan that suggest concerns regarding its influence on the patients’ quality of life and its cost effectiveness [46], and the question of whether tolvaptan administration should be recommended to patients is a worldwide issue. This current study showed that genetic mutations are associated with differences in renal function, which provides a rationale for considering aggressive intervention with tolvaptan in patients with *PKD1* mutations, especially in those with truncated mutations, as described above. This highlights the importance of genetic testing in clinical practice.

In the present study, there was a significant difference between *PKD1* or *PKD2* mutations and the percentages of low- or high-risk groups according to Mayo classification, as shown in Table 4. *PKD1* mutation was significantly associated with the high-risk group according to the Mayo classification. This suggests that *PKD1* mutations have an important prognostic relevance for renal function outcomes.

In this study, we found that the severity classification factors of the PROPKD score, hypertension < 35 years, and urological events < 35 years were not significant risk factors (Table 5). Sex has been reported as a risk factor for ADPKD in men [40,47]; however, in the present study, no significant correlation was found between sex and decreased renal function. In addition, urinalysis has previously been reported as a biomarker for predicting the progression of ADPKD. In particular, Messchendorp and Fick-Brosnahan et al. showed that urinary β2MG, urinary MCP-1, and proteinuria are useful predictive biomarkers of renal prognosis [16,17]. However, in the present study, no significant correlation was found between urinary protein and decreased renal function.

The present study had some limitations. First, there was a possibility of insufficient explanatory variables for risk factors in the multivariate analysis of ΔeGFR/year. Other explanatory variables that could have been included were blood markers such as hemoglobin, thrombocytes, blood sugar, uric acid, and high-density lipoprotein cholesterol [21,48,49,50,51]. Second, genetic testing is currently available in only a few facilities; therefore, the need for specialized genetic counseling and the cost of the test must be considered if the test is to be used as a popular and common test.

Nevertheless, in the present study, *PKD1* mutations (OR, 1.87; 95% CI, 1.11–3.16; *p* = 0.020) and an HtTKV ≥ 750 mL (OR, 1.67; 95% CI, 1.06–2.63; *p* = 0.029) were shown to be independent risk factors for ADPKD. The results of the multivariate analysis in the present study also indicated that each of these factors is an independent risk factor, suggesting that each factor is an important biomarker for predicting renal prognoses in Japanese patients (Table 5). In the Kaplan–Meier kidney survival plot, we found that the group of patients with a *PKD1* mutation showed significantly worse kidney survival compared to the group of patients with a *PKD2* mutation, and those with *PKD1* truncated mutations showed significantly worse kidney survival compared to those with *PKD1* non-truncated mutations (Figure 3). Therefore, in addition to kidney volume measurements, it is important to identify genetic mutation sites in Japanese patients with ADPKD.

## 5. Conclusions

In this large single-center prospective study that identified risk factors for renal function decline in Japanese patients with ADPKD, we showed that patients with *PKD1* mutations, especially truncated mutations, as well as those with increased HtTKV, are expected to show a rapid progression of renal dysfunction. Therefore, we showed that genetic mutations are useful biomarkers for predicting renal prognosis in ADPKD and that identification of genetic mutations by genetic testing can identify Japanese patients with ADPKD who are eligible for early treatment. We are hopeful that this study will lead to more widespread use of genetic testing for patients with ADPKD.

## Figures and Tables

**Table 1:** Irazabal equation coefficients for estimating future eGFR.

Irazabal Equation Coefficients for Estimating Future eGFR		
Variable	Description	Value
α	Intercept	21.18
β	Sex (reference is male)	−1.26
γ	Age at HtTKV0 (years)	−0.26
δ	eGFR at HtTKV0 (mL/min per 1.73 m^2^)	0.90
θb	Subclass 1B	0.58
θc	Subclass 1C	−1.14
θd	Subclass 1D	−1.93
θe	Subclass 1E	−6.26
ξ	Years from HtTKV0	−0.23
λ	Sex, years from HtTKV0	0.19
μ	Age at HtTKV, years from HtTKV0	−0.02
σc	Subclass 1C, years from HtTKV0	−2.63
σd	Subclass 1D, years from HtTKV0	−3.48
σe	Subclass 1E, years from HtTKV0	−4.78

Source: [23,32]. eGFR: estimated glomerular filtration rate, HtTKV: height-adjusted total kidney volume, HtTKV0: baseline height adjusted total kidney volume.

**Table 2:** Patient characteristics.

	Total	*PKD1* Truncated	*PKD1* Non-Truncated	*PKD2* Truncated	*PKD2* Non-Truncated	*p*-Value
Patients, n (%)	309 (100)	139 (45.0)	86 (27.8)	68 (22.0)	16 (5.2)	
Age, median (IQR)	48 (41–55)	46 (38–50)	46 (41–54)	52 (46–62)	54 (48–59)	<0.001
Sex						0.78
Female	176	79	49	37	11	
Male	133	60	37	31	5	
Height, m, median (IQR)	1.65 (1.58–1.72)	1.66 (1.60–1.73)	1.65 (1.60–1.72)	1.64 (1.56–1.70)	1.62 (1.57–1.66)	0.021
BMI, kg/m^2^, median (IQR)	22.0 (20.2–24.6)	21.7 (20.0–24.0)	22.7 (20.7–25.3)	21.9 (20.6–25.1)	23.0 (21.3–24.1)	0.304
TKV, mL, median (IQR)	1224.0 (808.0–1720.5)	1277.0 (840.0–1760.8)	1108.5 (755.2–1566.5)	1240.5 (809.8–1695.1)	1344 (900.8–3048.3)	0.016
HtTKV, mL/m, median (IQR)	748.0 (483.3–1002.2)	761.0 (525.5–1016.4)	694.1 (440.0–929.7)	753.9 (490.3–1033.6)	877.4 (575.6–1957.0)	0.011
ΔeGFR/year, mL/min/1.73 m^2^, median (IQR)	−3.10 (−5.69 to −1.0)	−3.65 (−6.39 to −1.35)	−3.41 (−5.69 to −1.66)	−2.04 (−5.01 to −0.60)	−2.22 (−5.00 to −0.58)	0.166
Hypertension before 35 years of age						0.118
Yes	41	24	12	4	1	
No	268	115	74	64	15	
Urologic event before 35 years of age						0.201
Yes	117	44	37	31	5	
No	192	95	49	37	11	
Mayo subclass						0.01
Class 1A	19	4	7	7	1	
Class 1B	103	39	29	29	6	
Class 1C	121	58	28	30	5	
Class 1D	54	29	20	2	3	
Class 1E	12	9	2	0	1	

Data are presented as either median (IQR) or n (%). IQR: interquartile range, BMI: body mass index, TKV: total kidney volume, HtTKV: height-adjusted total kidney volume, eGFR: estimated glomerular filtration rate, ΔeGFR/year: represents the 1-year change in eGFR calculated using the least-squares method based on the change in eGFR values before tolvaptan treatment.

**Table 3:** Subgroup analyses for ΔeGFR (*n* = 309).

	ΔeGFR/Year (mL/min/1.73 m^2^/Year)	*p*-Value
Age		0.334
<48	−3.41 [−5.88 to −1.03]	
≥48	−2.81 [−5.50 to −0.90]	
Sex		0.956
Female	−2.91 [−5.92 to −1.03]	
Male	−3.40 [−5.30 to −0.99]	
Height		0.867
<1.65	−2.86 [−5.7 to −1.24]	
≥1.65	−3.41 [−5.63 to −0.98]	
BMI		0.046
<22.0	−2.73 [−5.35 to −0.81]	
≥22.0	−3.61 [−6.09 to −1.38]	
HtTKV		0.020
<750	−2.64 [−5.12 to −0.83]	
≥750	−3.65 [−6.58 to −1.37]	
Mayo classification		0.035
1A, 1B	−2.38 [−4.98 to −0.98]	
1C, 1D, 1E	−3.61 [−6.39 to 1.15]	
Germline mutations		
PKD1	−3.50 [−6.31 to −1.40]	0.006
PKD2	−2.04 [−5.01 to −0.60]	
PKD1 truncated	−3.65 [−6.39 to −1.35]	0.955
PKD1 non-truncated	−3.41 [−5.69 to −1.66]	
PKD1 truncated (aged ≥ 65 years)	−6.56 [−6.58 to −4.80]	0.049
PKD1 non-truncated (aged ≥ 65 years)	−2.16 [−3.37 to −1.58]	
Hypertension before 35 years of age		0.207
Yes	−3.76 [−6.46 to −1.20]	
No	−2.95 [−5.57 to −0.99]	
Urologic event before 35 years of age		0.715
Yes	−3.03 [−5.62 to −0.80]	
No	−3.13 [−5.69 to −1.03]	

Data are presented as median (IQR). BMI: body mass index (kg/m^2^), HtTKV: height-adjusted total kidney volume (mL/m), eGFR: estimated glomerular filtration rate, ΔeGFR/year: represents the 1-year change in eGFR calculated using the least-squares method based on the change in eGFR values before tolvaptan treatment, aged > 65 years: the population over 65 years of age, IQR: interquartile range.

**Table 4:** Comparison of genetic mutations and factors that were significantly different in the subgroup analysis in the chi-square and Fisher’s exact tests.

	*PKD1*	*PKD2*	*p*-Value	*PKD1* Truncated	*PKD1* Non-Truncated	*p* Value	*PKD1* Truncated Aged ≥65 Years	*PKD1* Non-Truncated Aged ≥65 Years	*p*-Value
BMI			0.676			0.134			0.676
<22.0	115	40		77	38		2	5	
≥22.0	110	44		62	48		3	7	
HtTKV			0.626			0.253			0.626
<750	116	40		67	49		3	9	
≥750	109	44		72	37		2	3	
Mayo classification			0.015			0.127			0.131
1A, 1B	79	43		43	36		4	12	
1C, 1D, 1E	146	41		96	50		1	0	

BMI: body mass index (kg/m^2^), HtTKV: height-adjusted total kidney volume (mL/m).

**Table 5:** Odds ratios in the univariate and multivariate logistic regression analyses for renal dysfunction *.

	Univariate Analysis		Multivariate Analysis		Multivariate Analysis (PSM Data)	
	OR (95% CI)	*p*-Value	OR (95% CI)	*p*-Value	OR (95% CI)	*p*-Value
Age: ≥48 vs. <48 years	0.82 [0.52–1.28]	0.382				
Sex: male vs. female	1.01 [0.64–1.59]	0.968				
Height: ≥1.65 vs. <1.65 m	1.10 [0.70–1.72]	0.676				
BMI: ≥22.0 vs. <22.0	1.50 [0.96–2.35]	0.078				
HtTKV: ≥750 vs. <750	1.62 [1.03–2.54]	0.027	1.67 [1.06–2.63]	0.029	2.44 [1.23–4.82]	0.011
*PKD1* vs. *PKD2*	1.81 [1.08–3.05]	0.025	1.87 [1.11–3.16]	0.020	2.58 [1.30–5.13]	0.007
*PKD1*: truncated vs. non-truncated	1.17 [0.68–2.00]	0.575				
Hypertension before 35 years of age	1.33 [0.69–2.58]	0.390				
Urologic event before 35 years of age	0.78 [0.49–1.26]	0.307				
U-pro	2.03 [0.97–4.24]	0.060				

* Renal function using ΔeGFR/year > 3.61 mL/min/1.73 m^2^/year as the cutoff value [29,30]. *BMI:* body mass index (kg/m^2^), HtTKV: height-adjusted total kidney volume (mL/m), U-pro: urine protein, OR: odds ratio, CI: confidence interval, PSM: propensity score matching.

## Data Availability

Data are available upon request due to privacy or ethical restrictions.

## Supplementary Materials

The following supporting information can be downloaded at: https://www.mdpi.com/article/10.3390/biom13071020/s1, Table S1: Genetic diagnosis; Table S2: The data abstracted using matched-pair analysis with propensity score matching.
